# The Pre-Twin Screen Consortium proposal for fetal structural anomalies evaluation across all three trimesters in twin pregnancies

**DOI:** 10.1007/s00404-025-08044-0

**Published:** 2025-05-05

**Authors:** R. Svirsky, R. Maymon, N. Kugler, A. Orenstein, N. Z. Sharon, A. Sharabi-Nov, H. Meiri, M. Maslenko, R. Brown, H. Portillo-Rodrigue, A. Goncé, M. Bennasar, I. Matas, A. Geipel, A. Walter, C. Simonini, B. Strizek, E. Bevilacqua, E. Torcia, K. O. Kagan, J. Steiner, T. Lennartz, A. Bauer, K. H. Nicolaides, A. Borrell, the PreTwin Screen Consortium

**Affiliations:** 1 Department of Obstetrics and Gynecology, Shamir (Assaf Harofeh) Medical Center, Zerifin, Israel; 2 Faculty of Medicine and Health Sciences, Tel Aviv University, Tel Aviv, Israel; 3 Medical Genetic Unit, Department of Obstetrics and Gynecology, Samson Assuta Ashdod University Hospital, Ashdod, Israel; 4 School of Medicine, Faculty of Medicine and Faculty of Health Sciences, Ben-Gurion University of the Negev, Beer Sheva, Israel; 5 Tel Hai Academic College, Israel and Ziv Medical Center, Safed, Israel; 6 Division of Maternal Fetal Medicine, Department of Obstetrics & Gynaecology, McGill University Health Centre, Montreal, Canada; 7 Hospital Clínic of Barcelona, BCNatal, Materno-Fetal Medicine Center, Barcelona, Spain; 8 Department of Obstetrics and Prenatal Medicine, University Hospital Bonn, Bonn, Germany; 9 Department of Obstetrics and Gynecology, Fondazione Policlinico Universitario Agostino Gemelli IRCCS, Rome, Italy; 10 Maternal Fetal Medicine Unit, Department of Obstetrics and Gynecology, University Hospital Tubingen, Tubingen, Germany; 11 Fetal Medicine Research Institute, King’s College Hospital, London, UK

**Keywords:** Twin pregnancy, First-trimester scan, Malformations, Third-trimester scan, Detection rate

## Abstract

**Purpose:**

In singleton pregnancies, routine ultrasound examinations in each trimester improves the diagnosis of fetal abnormalities and their management. In this study, we examine twin pregnancies and report on the detection of fetal anomalies in each trimester and postnatally as well as on the type of anomalies and their prevalence.

**Methods:**

This prospective multicenter study enrolled pregnant women with dichorionic (DC) and monochorionic diamniotic (MCDA) twins at gestational weeks (GA) 11 + 0 to 13 + 6 in six medical centers (two in Germany, and one each in Spain, Canada, Italy, and Israel). Pregnancies were dated by the crown-rump length of the larger twin. Detailed scans were performed according to the guidelines of the International Society of Ultrasound in Obstetrics and Gynecology (ISUOG) at enrollment at gestational week (GA) 11 + 0 to 13 + 6, and at GA 20 + 0 to 22 + 6 and GA 28 + 0 to 32 + 6. Neonatal examinations were conducted in each case. Malformations were confirmed after delivery or postmortem in cases of fetal death or termination of pregnancy (TOP).

**Results:**

Of the 649 twin pregnancies (1298 live fetuses) at enrollment, there were 436 (70.6%) DC and 182 (29.4%) MCDA pregnancies. In total, 1168 babies were liveborn to 618 mothers (808 DC and 360 MCDA twins) after excluding cases lost to follow-up and TOPs. Anomalies were identified in 4.1% of the fetuses (48/1168). Of the 48 fetuses with anomalies, 17 (35.4%) were identified in the first, 21 (43.8%) in the second, and 6 (12.5%) in the third trimester. Additional 4 (8.3%) were identified postnatally. Of the anomalies, 37 were in fetuses from DC twins (4.57%), and 11 (3.05%) in MCDA twins.

**Conclusion:**

We demonstrate the utility of detailed fetal scans in twin pregnancies in all three trimesters. First- and second-trimester diagnosis enables genetic counseling and testing, and informed parental decisions of pregnancy management. In addition to alerting the parents to the presence of fetal anomalies, third-trimester scans enhanced delivery planning and newborn care.

**Supplementary Information:**

The online version contains supplementary material available at 10.1007/s00404-025-08044-0.

## What does this study add to the clinical work

**Table:** 

Overall, structural anomalies were identified in 4.1% of fetuses from twin pregnancies, including 35.4%, 43.7%, 12.5%, and 8.3%, detected in the first, second, and third-trimester scans and after birth, respectively. The findings demonstrate the necessity for three systematic ultrasound examinations in pregnancy, one in each trimester for the diagnosis of fetal defects to enable further testing, consultation, and reaching informed parental decision on the pregnancy continuation when discovered early in pregnancy and improved delivery planning and post-partum care when discovered late.	

## Introduction

The traditional approach to the diagnosis of fetal abnormalities, both in singleton and in twin pregnancies, is to perform two routine ultrasound scans in pregnancy, the first one at around 12 weeks’ gestation and the second at around 20 weeks [1–6]. There is now increasing evidence that a routine anomaly scan should also be offered in the third trimester [7, 8]. Twin pregnancies, especially monochorionic (MC) twins, have an increased prevalence of fetal malformations [3]. In MC twin pregnancies, there is twice as high a risk as in dichorionic (DC) twins of at least one of the fetuses to be affected by a major midline defect, such as anencephaly, facial cleft, cardiac anomalies, exomphalos, and spina bifida. Furthermore, in MC twins, there are specific abnormalities that are unique for such fetuses, such as the twin-reversed-arterial-perfusion (TRAP) sequence.

First- and second-trimester diagnosis of fetal defects enables the undertaking of all necessary further investigations and counseling for informed decisions by the parents on further pregnancy management. Third-trimester scans can detect late-onset anomalies and improve the delivery plans and post-partum care as well as alerting the parents of the presence of fetal malformations. Additionally, in some countries, late termination is allowed.

The objective of this multinational, multicenter study is to investigate the prevalence and types of fetal abnormalities identified by three routine ultrasound examinations (one in each trimester) in twin pregnancies and report the fetal abnormalities that are diagnosed only postnatally.

## Methods

### Study population

This was a prospective multicenter study of women with twin pregnancies in which both fetuses were alive at enrollment at 11 + 0 to 13 + 6 weeks’ gestation, as dated by the crown-rump length of the larger twin [9, 10]. There were two centers from Germany, and one each from Israel, Canada, Spain, and Italy.

Detailed ultrasound evaluation for the diagnosis of fetal abnormalities and assessment of fetal growth was offered to all women at 11 + 0 to 13 + 6 weeks’ gestation, 20 + 0 to 22 + 6 weeks, 28 + 0 to 32 + 6 weeks. Additional ultrasound examinations were performed in all twin pregnancies to assess fetal growth as recommended for all gestations and to look for twin-to-twin transfusion syndrome and TAPS in MCDA twins.

All participating centers implemented a comprehensive protocol outlining the minimum requirements for fetal ultrasound scans during each of the three trimesters of pregnancy. For the first trimester, the minimum requirements were derived from the “ISUOG Practice Guidelines: Performance of First-Trimester Fetal Ultrasound Scan.” The requirements for the second and third trimesters were based on the “Practice Guidelines for Performance of the Routine Mid-Trimester Fetal Ultrasound Scan.” (The minimal requirements for the scans are listed in Supplementary Table 1 for all the three trimesters) [4–6]. All centers were permitted to enhance these minimum requirement protocols. When the scan did not meet the minimum requirements, the centers were encouraged to invite the women for a follow-up scan to fulfill the full protocol. To improve diagnostic accuracy, when an anomaly was diagnosed, each patient was viewed by two experienced sonographers, and a post-session analysis of the ultrasound images was conducted in a team meeting at the end of each enrollment day to verify the diagnosis.

All scans were performed using commercially available ultrasound machines (General Electric E8/E10/E22, Toshiba (Canon) Aplio XG). In most cases, the scan was carried out transabdominally, but if visualization was poor, a transvaginal scan was conducted. Each examination in this study included a hands-on ultrasound evaluation by a fetal medicine specialist. We only included malformations as defined by the National Congenital Anomaly and Rare Disease Registration Service (NCARDRS) [11], and by the European Surveillance of Congenital Anomalies and Twins (EUROCAT) [12]. As this was an international study conducted in different centers and countries where local guidelines define major and minor malformations differently, and due to the importance of understanding the percentage of findings that may change the course of pregnancy in each trimester, we decided to include all abnormalities that were clinically considered to require enhanced surveillance and additional test methods (such as fetal MRI, chorionic villus sampling (CVS), amniocentesis) and parental consultation during the pregnancy and the early neonatal period were also included. This approach was associated with including cases of mild ventriculomegaly, cleft lip and palate, early fetal growth restriction (FGR), and increased NT, defined as NT ≥ 3.5 mm. “Soft markers” of chromosomal abnormalities, such as mild hydronephrosis (defined as an anteroposterior diameter of the renal pelvis of 4–7 mm and 7–9 mm in the second and third trimesters, respectively), echogenic cardiac foci, and single umbilical artery, were not counted as defects. We also did not include malformations, which we feel do not require further evaluation during pregnancy or early post-partum, such as small muscular ventricular septal defect [13, 14] or choroid plexus cyst. Complications specific to monochorionic diamniotic (MCDA) twins, such as twin-to-twin transfusion syndrome (TTTS) and other that are related to monochorionic placentation, including selective intrauterine growth restriction (sIUGR) and twin anemia-polycythemia sequence (TAPS), were not included in this publication. These topics will be covered in a subsequent analysis.

The master ethical approval for the study was obtained by Shamir (Assaf Harofeh) Medical Center Trial # 0043-20-ASF, Israel Ministry of Health Authorization # 202016632, and was subsequently approved in each participating center. All participants provided written informed consent to participate in the study. The protocol was registered on Clinicaltrial.gov with ID # NCT04595214.

### Statistical analysis

For descriptive statistics, the continuous variables are presented as medians and interquartile ranges [IQR], and the categorical variables as frequencies (*n*) and percentages. The analyses were performed using SPSS software (IBM SPSS Statistics for Windows, Version 28.0. Armonk, NY: IBM Corp). A *p* value of < 0.05 was considered statistically significant.

## Results

### Cohort characteristics

**Fig. 1:**
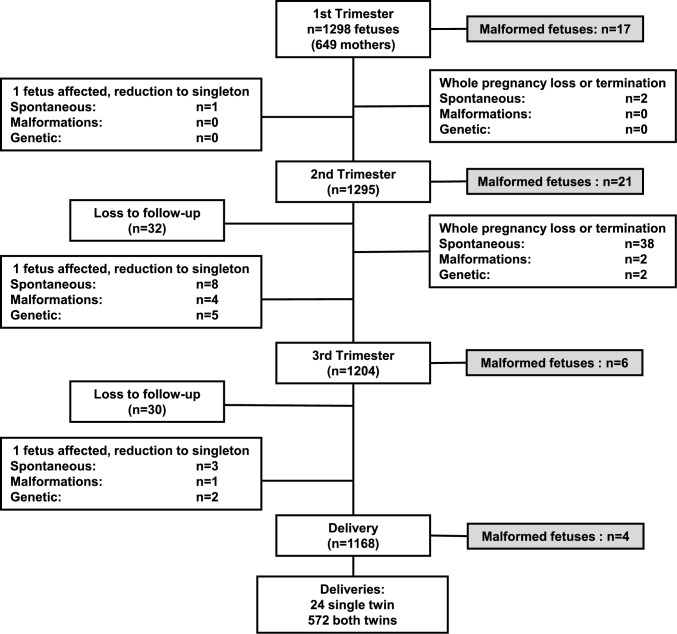
Flow chart of the study population

Enrollment in the study started in December 2020 and ended in August 2023, and the last woman delivered in February 2024. We enrolled 649 women (1298 fetuses) (Fig. 1).

There were 62 fetuses (31 mothers) who were lost to follow-up after the second-trimester scan and 60 fetuses (30 mothers) after the third-trimester scan. There were 20 pregnancies (40 fetuses) with spontaneous death of both fetuses (1 after the first-trimester scan and 19 after the second-trimester scan). In addition, there were two pregnancies (four fetuses) where pregnancy termination was carried out, one due to structural malformations and one due to genetic anomalies.

Spontaneous death of one twin occurred in 12 cases (1 after the first-trimester scan, 8 after the second-trimester scan, and 3 after the third-trimester scan). In addition, there were 12 cases of selective reduction of 1 twin due to structural or genetic anomalies (9 after the second-trimester scan and 3 after the third-trimester scan). There were 1168 live births, 1144 as twins, and 24 as singletons, 360 from MCDA and 808 from DC pregnancies.

Table 1 summarizes the characteristics and pregnancy outcomes of the included population. Women were enrolled at a median of 12.6 weeks’ gestation and their median age was 34.1 years. In 12.5% of the cases, the BMI was > 29 kg/m^2^. Most women (89%) were of white ethnicity, and 57.4% were nulliparous. Most pregnancies (70.6%) were DC. In a high proportion of cases (40.3%), conception was through assisted reproduction technologies. The median gestational age at delivery was 36.6 weeks, indicating that, as expected, about 50% of the twins were born before term. The median gestational age-adjusted birthweight percentile for DC twins (47.2%, IQR 41.6%–52.7%) was significantly higher (*p* < 0.001) than in MCDA twins (43.4%, IQR 34.8%–49.9%) [15, 16].

**Table 1:** Cohort characteristics (618 mothers, 1168 newborns)

Enrollment	
Maternal age, years, Me (IQR)	34.1 (30.9–37.0)
Body mass index, kg/m^2^, Me (IQR)	24.5 (21.8–28.7)
Ethnicity, *n* (%)	
White	550 (89.0)
Non-white	68 (11.0)
Nulliparous, *n* (%)	355 (57.4)
Conception method, *n* (%)	
Spontaneous	369 (59.7)
Assisted reproductive technologies	249 (40.3)
Chorionicity, *n* (%)	
Dichorionic	436 (70.6)
Monochorionic diamniotic	182 (29.4)
Gestational age, weeks, Me (IQR)	
1 st trimester	12.6 (12.1–13.0)
2nd trimester	21.1 (20.6–22.0)
3rd trimester	29.0 (28.4–29.7)
**Delivery**	
Gestational age at delivery, weeks, Me (IQR)	36.6 (35.0–37.3)
Delivery of live MCDA twins, n (%)	360 (30.8)
Delivery of live DC twins, n (%)	808 (69.2)
Delivery of one live twin, n (%)	24 (2.1)
Delivery of two live twins, n (%)	1144 (97.9)
Newborn gender female, n (%)	569/1168 (48.7)
Twin A birthweight, grams, Me (IQR)	2420 (2110–2700)
Twin B birthweight, grams, Me (IQR)	2380 (2060–2674)
Birthweight of DC twins, grams, Me (IQR)	2452 (2165–2740)
Birthweight of MCDA twins, grams, Me (IQR)	2258 (1860–2540)
Birthweight percentile in DC twins, Me (IQR)	47.3 (41.6–52.7)
Birthweight percentile in MCDA twins, Me (IQR)	43.4 (34.8–49.0)

Values are given as median (Me) and interquartile range (IQR) or *n* (%)

*DC* dichorionic, *MC* monochorionic diamniotic

### Malformations

Table 2 summarizes that in total, there were 4.1% (48/1168) abnormalities identified in this study, including 17 (35.4%) in the first-trimester, 21 (43.8%) in the second-trimester, and 6 (12.5%) in the third-trimester and 4 (8.3%) were detected postnatally. In the first-trimester group, 14 were from DC twins and 3 from MCDA twins. In the second-trimester group, 13 were from DC twins and 8 from MCDA twins. In the third-trimester group, 5 were from DC twins and 1 from MCDA twins. Postnatally, two cases of inguinal hernia and two cases of hypospadias were found. All other neonates had no detectable anomalies. Additionally, the proportion of malformations in MCDA twins was smaller than that of DC twins. Accordingly, of the 48 cases, 37 were DC twins (4.57%), and only 11 were MCDA twins (3.05%).

**Table 2:** Individual twin fetuses abnormalities diagnosed during pregnancy and postnatally

Malformations/anomalies	Genetics	Chorion	Outcome
**First-trimester scan (11**^**+0**^** to 13**^**+6**^** weeks, 17 cases, 11 major)**			
• Hypoplastic right heart, cervical cyst, abnormal V sign, ductus venosus reverse flow, FGR	• Low risk by cfDNA test	DC	• Fetal death 13 w
• High NT (3.9 mm), Tetralogy of Fallot,	• Trisomy 21	DC	• Selective Termination 16 w
• Unilateral renal agenesis and contralateral large echogenic kidney	• Low risk by cfDNA test	DC	• Selective Termination 18 w
• Omphalocele, scoliosis, renal agenesis	• Low risk by cfDNA test	DC	• Selective Termination 15 w
• Tetralogy of Fallot	• Normal CMA	DC	• Both liveborn
• Bilateral cleft lip and palate, single umbilical artery	• Trisomy 13	DC	• Selective Termination 19 w
• Ventriculomegaly*	• Normal CMA	MC	• Selective Termination 14 w
• Low urinary tract obstruction	• Low risk by cfDNA test	DC	• Both liveborn
• Hypoplastic left heart syndrome	• Low risk by cfDNA test	DC	• Both liveborn
• Early severe FGR	• Low risk by cfDNA test	MC	• Both liveborn
• Diaphragmatic hernia	• Normal WES	DC	• Both liveborn
• Diaphragmatic hernia		MC	• Selective Termination 18 w
• Unilateral clubfoot	• Low risk by cfDNA test, Normal CMA	DC	• Both liveborn
• High NT (3.8 mm)*	• Trisomy 21 by cfDNA test, Ebstein anomaly at 16 w	DC	• Both liveborn
• High NT (5.9 mm)*	• Low risk by cfDNA test, Monosomy X	DC	Selective Termination 18 w
• Two cases of high NT (3.8 and 5.8 mm) *	• Low risk by cfDNA test	DC 2	• Two both liveborn
**Second trimester scan (19 + 0 to 25 + 6 weeks, 21 cases, 18 major)**			
• Six cases of clubfoot, two bilateral	• All low risk by cfDNA test, three normal CMA	DC 5, MC 1	• In all six, both liveborn
• Three cases of severe FGR*	• All low risk by cfDNA test, two normal CMA	DC 3	• Two both liveborn, one IUFD at 30w and one liveborn
• Two cases of agenesis/dysgenesis of the corpus callosum	• Both low risk by cfDNA	DC 1, MC 1	• DC selective termination, MC both liveborn
• Two cases of pelvic kidney	• Both low risk by cfDNA test	MC 2	• Both liveborn
• Hypertrophic right ventricle of the fetal heart	• Low risk by cfDNA test	MC	• Both liveborn
• Transposition of great arteries	• Low risk by cfDNA test	DC	• Both liveborn
• Atrioventricular canal	• Trisomy 21	DC	• Both liveborn
• Tetralogy of Fallot	• Low risk by cfDNA test	MC	• Both liveborn
• Abnormal genitalia	• normal CMA	MC	• Both liveborn
• Multiple malformations (micrognathia + pyelectasis + ventricular septal defect)	• WES: 7q.221q22.3 deletion)	DC	• Selective Termination 29 w
• Cleft lip and palate	• Low risk by cfDNA test	DC	• Both liveborn
• Unilateral dysplastic kidney	• Normal CMA	MC	• Both liveborn
**Third-trimester scan (28 + 0 to 30 + 6 weeks, 6 cases, 2 major)**			
• Two cases of mega cisterna magna, one with ventriculomegaly and one diagnosed with inguinal hernia after birth*	• Low risk by cfDNA test	DC 1, MC 1	• Two both liveborn
• A case of mild ventriculomegaly*	• Low risk by cfDNA test Abnormal WES	DC 2	• Two both liveborn
• A case of mild ventriculomegaly diagnosed postnatally with dysgenesis of the corpus callosum*	• Low risk by cfDNA test • Abnormal WES	DC 2	• Two both liveborn
• Metopic craniosynostosis	• Normal CMA	DC	• Both liveborn
• Right aortic arch	• Low risk by cfDNA test	DC	• Both liveborn
**Postnatally (4 cases, 1 major)**			
• Two cases of inguinal hernia*		DC 2	• Two both liveborn
• Two cases of hypospadias* • dysgenesis of the corpus callosum		DC 1, MC 1	• Two both liveborn

In total there were 48 abnormalities that required further testing and prenatal counseling (4.1% of 1168) including 17 (35.4%) in the first trimester, 21 (43.8%) in the second trimester, 6 (12.5%) in the third trimester, and 4 (8.3%) detected postnatally

The aster risk marked abnormalities that are the ones not considered major in some centers in this study and in many centers outside this study. Accordingly, the asterisk marked excluded cases of FGR, mild ventriculomegaly, high NT as a single finding, mega cisterna, inguinal hernia, and hypospadias. In this narrower definition, the number of anomalies was 35 (3% of 1168) of the fetuses or neonates, including 11 (31.4%) in the first trimester, 18 (51.4%) in the second trimester, 2 (5.7%) in the third trimester, and 1 (2.9%) detected postnatally

*DC* dichorionic, *MC* monochorionic diamniotic, *cfDNA* cell-free DNA, *CMA* chromosomal microarray, *WES* whole exome sequencing, *NT* nuchal translucency, *FGR* fetal growth restriction, *NT* nuchal translucency

After subtracting certain malformations such as fetal growth restriction (FGR), mild ventriculomegaly, high nuchal translucency (NT), mega cisterna magna, inguinal hernia, and hypospadias that might not be universally recognized as major anomalies, the number of malformations is reduced to 33 representing 2.8% of the 1168 cases (Table 2). Using this approach, 12 (36.3%) major abnormalities were detected in the first trimester, 18 (54.5%) in the second trimester, 2 (6.1%) in the third trimester, and one (3%) was identified postnatally.

A secondary analysis was conducted to investigate why certain malformations that were missed during the first trimester were identified in the evaluation conducted during the second trimester. Our findings revealed that the missed detections could not be attributable to high BMI, lack of sonographer experience, or similar confounding variables. Instead, the missed malformations in the first trimester were associated with first trimester examination at the first half of the 11^th^ gestational week. The secondary analysis also indicated that small fetal size (fetal size < the 5^th^ percentile for gestational week) was typically associated with these cases.

## Discussion

### Main findings

This prospective, multicenter, and multinational study in twin pregnancies has demonstrated the importance of three routine anomaly scans, one in each trimester, for the diagnosis of fetal abnormalities. First- and second-trimester diagnosis of fetal defects enables the undertaking of all necessary further investigations and counseling for informed decisions by the parents on further pregnancy management. Third-trimester scans that detect late-onset abnormalities, improve the planning of delivery of these fetuses and their post-partum care. Additionally, in some countries, the option of late termination is available.

### Comparison with findings of previous studies

In three previous studies, in 495, 1,084, and 6,366 twin pregnancies, the proportion of defects detected in the first trimester was 13%, 27%, and 36.5%, respectively [3, 17, 18]. The proportion of the total number of abnormalities that was detected in the first trimester reported here is 35.4%, which is consistent with the study of Syngelaki et al. [3]*.* In relation to the types of defects that are detected at 11–13 weeks, the results are consistent with those published in previous studies in singleton pregnancies, which highlighted that defects can essentially be divided into those that should be always detectable, those that are potentially detectable, and those that are undetectable [2].

When detection in the three trimesters is compared, a previous study in a series of 1720 singleton pregnancies reported that the prevalence of fetal malformations was 1.7%, and 27.6%, 53.8%, and 18.6% were detected in the first-, second-, and third-trimesters, respectively [2]. In our study of twins, the prevalence of malformations at 4.1% was considerably higher than in singletons, and the respective rates for detection in the first-, second- and third-trimesters were 35.4%, 43.7%, and 12.5%. Therefore, in both singleton and twin pregnancies > 80% of the malformations are detected during the first- and second-trimester scans.

A study of 52,400 singleton pregnancies undergoing a routine ultrasound examination at 36 weeks’ gestation reported that 1.9% of fetuses had malformations and 7.4% of these were first detected in the third trimester [7], which is similar to the 8.3% observed in our study. There are no previous third-trimester cohort studies investigating defects in twin pregnancies.

Interestingly, a recent study from Israel evaluating the sequence of events leading to late abortion for fetal indications reported that in about 60% of abortions after 34 weeks gestation, the types of fetal anomalies present were not detectable prior to the third trimester. [19].

In a secondary analysis, we found that certain risk factors were associated with abnormalities that were initially missed in the first trimester but later identified in the second trimester. These factors include enrollment in the 11^th^ week of gestation and the presence of small fetuses (growth at or below the 5^th^ percentile). Based on our findings, we recommend that the first-trimester anatomical evaluation should begin at the 12^th^ week of gestation, preferably in its midpoint for small fetuses, as previously suggested by Yagel et al. [20].

Among the six fetuses with malformations identified only in the third trimester, five had what are considered true “late onset” malformations, which include mega cisterna magna, mild ventriculomegaly, and metopic craniosynostosis. There was an additional malformation—a right aortic arch—which was probably missed during the second-trimester scan. It is important to note that abnormalities identified only after birth included malformations that are rarely detected prenatally, such as inguinal hernia and hypospadias. These conditions have effective treatments and a minimal impact on children’s outcomes. This indicates that no significant malformations were missed when scans from all three trimesters were combined.

### Clinical implications

This study assessed the added value of first- and third-trimester anatomical scans in twin pregnancies in addition to the traditional second-trimester scan for the diagnosis of fetal abnormalities. First-trimester diagnosis allows more time for multidisciplinary parental counseling, consideration for advanced genetic testing (CMA, WES), and reaching an informed parental decision regarding subsequent pregnancy management. If the parents desire termination of the entire pregnancy or selective reduction, a procedure conducted at early gestation is both physically and psychologically less traumatic. Third-trimester diagnosis of fetal abnormalities is useful in selecting the place, method and time of delivery which could potentially improve perinatal outcomes.

### Strengths and weaknesses

A major strength of our study is that it is multicenter and multinational, making the results generalizable. The study was prospective and undertaken in centers with expertise in Fetal Medicine, following a standardized protocol that combined ultrasound with genetic evaluation in each trimester. Furthermore, prospective and systematic data collection and recording during pregnancy, at delivery, and after birth strengthened the validity of the study’s results.

Although the number of fetuses examined was more than one thousand, examination of larger cohorts is necessary to define the true prevalence and types of major malformations that can be identified in the three trimesters of twin pregnancies.

This study defined abnormalities as “abnormalities requiring enhanced surveillance, additional testing methods, and parental counseling.” We adopted this approach as we feel that it is important to report the percentage of findings that may change the course of pregnancy management in each trimester. Additionally, this approach established a common ground among all participating centers and countries, across different guidelines and rules for pregnancy managements and termination.

We recognize that in many centers worldwide, including some of ours, the standard definition is “major abnormalities.” Conditions such as fetal growth restriction (FGR), mild ventriculomegaly, a high nuchal translucency (NT) as an isolated finding, mega cisterna magna, inguinal hernia, and hypospadias are often not counted as “major” abnormalities. However, a universal definition of “major abnormalities” has not yet been developed. Given that this is a multinational and multicenter study operating under various standards, we believe that a definition that considers the clinical need for additional testing and parental counseling is a reasonable one and provides a more unified ground for prenatal management. Table 2 includes both our definition and of the ones considered “non-major anomalies.”

One limitation of our research was the absence of a separate analysis differentiating between low-risk and high-risk groups. High-risk cases, such as those with increased nuchal translucency (NT) or a previous history of malformations or repeated pregnancy loss, were underrepresented. Consequently, there were too few high-risk cases to conduct a statistically valid separate accuracy analysis.

We did not exclude high-risk pregnancies (other than twins that developed twin-to-twin transfusion syndrome (TTTS) or other complications related to monochorionic placentation, such as selective intrauterine growth restriction (sIUGR) or twin anemia-polycythemia sequence (TAPS) as the project aimed to evaluate the procedures of managing all twin pregnancies; low and high risk included.

Of the evaluable twins (1168), 48 abnormalities (4.1%) were identified. One of the study limitations is related to our lack information about potential additional abnormalities in those that were lost to follow-up, and in cases of spontaneous loss of one or both twins, who avoided or could not provide samples for post mortem analysis.

## Conclusion

In this prospective multicenter and multiethnic study of twin pregnancies, we demonstrated the feasibility and importance of the first- and third-trimester anomaly scans, in addition to the standard second-trimester anatomical evaluation. Such findings are consistent with the results of previous studies reporting on the diagnosis of fetal abnormalities in the first and third trimesters of pregnancy [1–8].

## Supplementary Information

Below is the link to the electronic supplementary material.

Supplementary file1 (DOCX 17 KB)

## Data Availability

The data that support the findings of this study are available upon reasonable request from the corresponding author.

## Abbreviations

BMI: Body mass index
cffDNA: Circulating free fetal DNA
CMA: Chromosomal microarray
CRL: Crown-rump length
CVS: Chorionic villus sampling
DC Twins: Dichorionic twins
FGR: Fetal growth restriction
IQR: Inter quartile range
MCMA twins: Monochorionic monoamniotic twins
MCDA Twins: Monochorionic diamniotic twins
NT: Nuchal translucency
sIUGR: Selective intrauterine growth restriction
TAPS: Twin anemia-polycythemia sequence
TRAP Sequence: Twin-reversed-arterial-perfusion sequence
WES: Whole exome sequencing
