# Supplementary material for: The Pre-Twin Screen Consortium proposal for fetal structural anomalies evaluation across all three trimesters in twin pregnancies
Source: Arch Gynecol Obstet. 2025 May 5;312(2):537–45. doi: 10.1007/s00404-025-08044-0 (PMC12334499; doi:10.1007/s00404-025-08044-0)
Supplement: Supplementary file 1 — Supplementary file1 (DOCX 17 KB) [file 404_2025_8044_MOESM1_ESM.docx]

**SUPPLEMENT 1: SCAN MINIMAL REQUIREMENTS**

**First trimester minimal requirements:**

| **Present / Normal** | **Organ /**  **anatomical area** |
| --- | --- |
| Cranial bones, midline falx, choroid-plexus-filled ventricles | Head |
| Nuchal translucency thickness | Neck |
| Eyes with lens, nasal bone, normal profile/mandible, intact lips | Face |
| Vertebrae (longitudinal and axial), intact overlying skin | Spine |
| Symmetrical lung fields. No effusions or masses | Chest |
| Cardiac regular activity, four symmetrical chambers | Heart |
| Normal cord insertion | Abdominal wall |
| Four limbs each with three segments. Hands and feet with normal orientation | Extremities |
| Three-vessels | Umbilical Cord |

**Second and third trimester minimal requirements:**

| **Present / Normal** | **Organ /**  **anatomical area** |
| --- | --- |
| Intact cranium, cavum septi pellucidi, midline falx, thalami, cerebral ventricles, cerebellum, cisterna magna | Head |
| Both orbits present, median facial profile, upper lip intact | Face |
| Absence of cysts or masses | Neck |
| Normal shape/size of chest and lungs, aortic and pulmonary outflow tracts, no evidence of diaphragmatic hernia. | Chest/Heart |
| Stomach in normal position, bowel not dilated, kidneys both present, cord insertion site is listed | Abdomen |
| Cardiac regular activity, Four symmetrical chambers | Heart |
| Normal cord insertion | Abdominal wall |
| No spinal defects or masses, four extremities are present, normal relationships | Skeletal |
| Position, No masses present, presence of accessory lobe | Placenta |
| Male or female | Genitalia |
| Three-vessel cord | Umbilical Cord |
